# Procollagen type 1 N-terminal propeptide is associated with adverse outcome in acute chest pain of suspected coronary origin

**DOI:** 10.3389/fcvm.2023.1191055

**Published:** 2023-09-04

**Authors:** Thomas Andersen, Thor Ueland, Pål Aukrust, Dennis W.T. Nilsen, Heidi Grundt, Harry Staines, Volker Pönitz, Frederic Kontny

**Affiliations:** ^1^ Department of Anesthesiology, Stavanger University Hospital, Stavanger, Norway; ^2^ Department of Clinical Science (K2), University of Bergen, Bergen, Norway; ^3^ Research Institute of Internal Medicine, Oslo University Hospital Rikshospitalet, Oslo, Norway; ^4^ Thrombosis Research Centre (TREC), Department of Clinical Medicine, UiT—The Arctic University of Norway, Tromsø, Norway; ^5^ Institute of Clinical Medicine, Faculty of Medicine, University of Oslo, Oslo, Norway; ^6^ Section of Clinical Immunology and Infectious Diseases, Oslo University Hospital Rikshospitalet, Oslo, Norway; ^7^ Department of Cardiology, Stavanger University Hospital, Stavanger, Norway; ^8^ Department of Clinical Science, University of Bergen, Bergen, Norway; ^9^ Department of Pulmonology, Stavanger University Hospital, Stavanger, Norway; ^10^ Sigma Statistical Services, Balmullo, United Kingdom; ^11^ Drammen Heart Centre, Drammen, Norway

**Keywords:** biomarkers, acute coronary syndromes, mortality/survival, risk factors, extracellular matrix, collagen

## Abstract

**Background:**

Extracellular matrix (ECM) is an integral player in the pathophysiology of a variety of cardiac diseases. Cardiac ECM is composed mainly of collagen, of which type 1 is the most abundant with procollagen type 1 N-terminal Propeptide (P1NP) as a formation marker. P1NP is associated with mortality in the general population, however, its role in myocardial infarction (MI) is still uncertain, and P1NP has not been investigated in acute chest pain. The objective of the current study was to assess the role of P1NP in undifferentiated acute chest pain of suspected coronary origin.

**Methods and results:**

813 patients from the Risk in Acute Coronary Syndromes study were included. This was a single-center study investigating biomarkers in consecutively enrolled patients with acute chest pain of suspected coronary origin, with a follow-up for up to 7 years. Outcome measures were a composite endpoint of all-cause death, new MI or stroke, as well as its individual components at 1, 2, and 7 years, and cardiac death at 1 and 2 years. In multivariable Cox regression analysis, quartiles of P1NP were significantly associated with the composite endpoint at 1 year of follow-up with a hazard ratio for Q4 of 1.82 (95% CI, 1.12–2.98). There was no other significant association with outcomes at any time points.

**Conclusion:**

P1NP was found to be an independent biomarker significantly associated with adverse clinical outcome at one year in patients admitted to hospital for acute chest pain of suspected coronary origin. This is the first report in the literature on the prognostic value of P1NP in this clinical setting.

**Clinicaltrials.ygov Identifier:**

NCT00521976.

## 1. Introduction

Chest pain is a common complaint in emergency departments with causes that range from innocent muscle pain to life-threatening diseases. The most common serious cause of chest pain is acute coronary syndrome (ACS), which in the aftermath induces a remodeling process with both positive adaptation and scar healing, but also with a potential for development of heart failure (HF) (1). This remodeling process includes both changes in the cellular parts of the tissue (i.e., cardiomyocytes and cardiac fibroblasts), as well as in the extracellular matrix (ECM). ECM is not only a protein-rich scaffolding for the cells, but is also increasingly recognized as an important regulator of cellular responses (1). Thus, regulation and dysregulation of ECM homeostasis is a principal component of both cardiac health and disease development. The homeostasis of ECM remodeling and disease development is complex. However, individual components of the ECM have been found to be associated with adverse outcome and even have potential as therapeutic targets (2).

Fibrillar collagen is the predominant component of cardiac ECM, with ∼85% being collagen type 1, conferring tensile strength (3, 4). Cardiac fibroblasts are the main producer of collagen within the heart. It is secreted as procollagens that are subsequently cleaved to produce mature collagen proteins by splitting off both C-terminal and N-terminal ends. By-products of the procollagen maturation, such as Procollagen type 1 N-terminal propeptide (P1NP), has been used as biomarkers of collagen synthesis in various disorders that involve ECM remodeling as a pathological feature (5).

The prognostic value of P1NP has been investigated in myocardial infarction (MI) and as a potential marker of ischemic heart disease in apparently healthy individuals, but the results are mixed, with some series showing association with risk, while others found no association (6–8). Only two small studies have investigated P1NP in patients with chest pain, both cohorts containing patients with stable symptoms electively referred for further diagnostic work-up (9, 10). No series have so far studied this biomarker in patients presenting to the emergency department with acute chest pain with suspected ACS.

In this study, we wanted to investigate the prognostic value of P1NP with respect to adverse clinical outcome including morbidity and mortality, in an undifferentiated acute chest pain cohort.

## 2. Material and methods

### 2.1. Population

This study is an extension of the single center prospective cohort study Risk in Acute Coronary Syndrome (RACS—ClinicalTrials.gov Identifier: NCT00521976). Consecutive patients presenting to Stavanger University Hospital, Norway, with acute chest pain suggestive of ACS were included from November 2002 to October 2003 (11). Exclusion criteria were age <18 years, incapacity or unwillingness to give consent, or prior inclusion in the study. Patients were followed for up to 7 years, with contact at 30 days and 6, 12, 24, and 84 months to obtain survival status, date and cause of death, as well as other clinical data (12). The patient's general practitioner or nursing home were contacted if interviews were not possible. Hospital records were used for data confirmation. Written informed consent was given by all patients. The trial adhered to the Helsinki Declaration and was approved by the Regional Board of Ethics and Norwegian health authorities.

### 2.2. Endpoints

The main outcome measure of the current study was a combination of all-cause death, new MI or stroke within 1 year. Other outcomes included the combined endpoint at 2— and 7-years follow-up, as well as its individual components at 1, 2, and 7 years. Cardiac death at 1 and 2 years, and the combination of cardiac death, MI or stroke at 1 and 2 years were also analyzed.

Cardiac death was defined as death after a definite MI or chest pain lasting more than 20 min, or a history of ischemic heart disease and no other clear cause of death. ACS incorporates ST-elevation MI (STEMI), Non-ST-elevation MI (NSTEMI), and unstable angina pectoris (UAP). MI was defined as symptoms of coronary ischemia with or without ST-segment changes on ECG and a typical rise and fall of Troponin T (TnT) with levels ≥0.05 ng/ml. As opposed to NSTEMI, STEMI required ST-segment elevation. UAP was diagnosed in acute chest pain with clinically suspected ACS, with or without ST-segment changes and serial TnT levels below 0.05 ng/ml. All other cases were defined as non-ACS, and included conditions such as arrhythmia or unspecified chest pain. Prespecified analyses were performed in the TnT-positive subgroup. In these analyses a lower level (i.e., 0.01 ng/ml, the lower level of detection of the assay used) were chosen as cut-off to adjust for the diagnostic difference using a contemporary analytical assay.

### 2.3. Laboratory analyses

Venous blood samples were obtained immediately following admission to hospital. After centrifugation, Ethylenediaminetetraacetic Acid (EDTA) plasma and serum were stored at −80°C until analyses. Hematology and other baseline laboratory data were collected from the individual hospital records. Analysis of TnT, Brain Natriuretic peptide (BNP), and high-sensitivity C-Reactive Protein (hsCRP) have been described previously (13).

P1NP was measured in duplicate by an enzyme immunoassay (EIA) with matched antibodies from MyBioSource (RRID: AB_2927799, San Diego, CA, USA) in a 384-format using a combination of a SELMA (Jena, Germany) pipetting robot and a Biotek (Winooski, VT, USA) dispenser/washer. Absorption was read at 450 nm with wavelength correction set to 540 nm using an ELISA plate reader (Bio-Rad, Hercules, CA, USA). PINP intra- and inter-assay coefficients of variation were 6.9% and 8.9%, respectively.

### 2.4. Statistical analyses

Baseline characteristics are presented by quartiles of P1NP. Continuous variables are presented as median and interquartile range, and analyzed across quartiles of P1NP by the Kruskal–Wallis test. Categorical variables are presented as frequency and percentage, and analyzed across quartiles of P1NP by Chi-square tests. Biomarkers were analyzed as quartiles, except for TnT which was analyzed as a binary detectable variable (i.e., yes/no). Survival analyses are presented as Kaplan–Meier plots and tested using Log-Rank tests. Receiver Operator Characteristics (ROC)-curves and Area under ROC (AUROC) were estimated to test the ability of P1NP to predict outcome. Associations between P1NP and outcomes were assessed by both univariable Cox proportional hazards analysis and by a multivariable Cox analysis with a stepwise inclusion of common confounding variables. First, the multivariable Cox analysis was adjusted for significant confounding variables, thereafter P1NP was introduced in a second step. Significant confounding variables were selected from the baseline characteristics; age, sex, diabetes mellitus, hypertension (HT), current smoking, dyslipidemia, prior heart disease (Angina or MI), HF, prior medication (statins, beta-blockers, angiotensin converting enzyme inhibitors [ACEI] or angiotensin receptor blockers [ARB], diuretics and acetylsalicylic acid [ASA]), discharge diagnosis (STEMI/NSTEMI/UAP/Non-ACS), primary revascularization within 50 days of index event, as well as biomarkers (BNP, estimated Glomerular Filtration Rate [eGFR, By MDRD method], hsCRP and TnT). A two-sided *p*-value of <0.05 was considered statistically significant. Categorical variables in the model with >2 levels were tested both for the equality of the hazard ratio (HR) across levels, and for each level against the reference level. A likelihood ratio (LR)-test was conducted to assess if the addition of P1NP quartiles significantly improved the multivariable model. Only variables with *p* < 0.05 in both (for univariable analyses) or all three tests (for multivariable analyses) were considered significant. We included only cases with complete data in our analyses. Patients that did not experience the endpoint before our defined time-points of 1, 2 and 7years follow-up were censored in the respective analyses. We did not have any loss to follow-up and hence have no discontinuations in our data set. As this study is purely exploratory in origin, no adjustments were made for multiple comparisons and no power calculations were performed. We used all available samples from the biobank of the RACS study and the sample size was dictated by this. All statistical analyses were performed using SPSS version 25 (IBM, Armonk, New York, USA).

## 3. Results

P1NP measurements were available for 813 patients, and a flow chart for the study participants is shown in Supplementary Figure S1. The median level of P1NP was 42.4 ng/ml (32.1–55.4). There was no significant association between quartiles of P1NP and any of the baseline characteristics (Table 1). The composite endpoint of all-cause death, new MI or stroke occurred in 156 (19.2%) of the patients within the first year, and in 236 (29.0%) and 392 (48.2%) patients at 2 and 7 years, respectively. Frequencies for all outcomes are shown in Table 2.

**Table 1:** Baseline characteristics.

Characteristic		P1NP (ng/ml)					
		Quartile 1	Quartile 2	Quartile 3	Quartile 4	*P*-value	Total
		*n* = 203	*n* = 203	*n* = 204	*n* = 203		*n* = 813
		2.5–31.9	32.2–42.3	42.4–55.3	55.5–140.8		2.5–140.8
Demographics	Age, years, median (q1-q3)	73.0 (60.7–80.9)	73.4 (59.7–82.0)	72.2 (58.1–80.1)	70.3 (57.2–82.5)	0.686^†^	72.6 (59.0–81.1)
	Male, *n* (%)	116 (57.1)	123 (60.6)	134 (65.7)	126 (62.1)	0.358*	499 (61.4)
Comorbidities	Diabetes mellitus type I or II, *n* (%)	29 (14.3)	28 (13.8)	31 (15.2)	24 (11.8)	0.791*	112 (13.8)
	Hypertension, *n* (%)	90 (44.3)	84 (41.4)	78 (38.2)	89 (43.8)	0.582*	341 (41.9)
	Current smoking, *n* (%)	53 (26.1)	46 (22.7)	52 (25.5)	58 (28.6)	0.597*	209 (25.7)
	Dyslipidemia, *n* (%)	109 (53.7)	103 (50.7)	89 (43.6)	103 (50.7)	0.214*	404 (49.7)
	Prior MI or angina, *n* (%)	112 (55.2)	109 (53.7)	112 (54.9)	123 (60.6)	0.507*	456 (56.1)
	Prior heart failure, *n* (%)	52 (25.6)	55 (27.1)	49 (24.0)	65 (32.0)	0.298*	221 (27.2)
Medication Prior to admission	Statins, *n* (%)	67 (33.0)	71 (35.0)	73 (35.8)	72 (35.5)	0.937*	283 (34.8)
	Betablocker, *n* (%)	72 (35.5)	78 (38.4)	66 (32.4)	77 (37.9)	0.563*	293 (36.0)
	ACEI/ARB, *n* (%)	67 (33.0)	66 (32.5)	67 (32.8)	76 (37.4)	0.687*	276 (33.9)
	Diuretics, *n* (%)	50 (24.6)	66 (32.5)	63 (30.9)	73 (36.0)	0.094*	252 (31.0)
	ASA, *n* (%)	74 (36.5)	77 (37.9)	77 (37.7)	82 (40.4)	0.873*	310 (38.1)
Index diagnosis						0.279*	
	UAP, *n* (%)	17 (8.4)	15 (7.4)	24 (11.8)	18 (8.9)		74 (9.1)
	NSTEMI, *n* (%)	58 (28.6)	59 (29.1)	60 (29.4)	58 (28.6)		235 (28.9)
	STEMI, *n* (%)	38 (18.7)	26 (12.8)	33 (16.2)	21 (10.3)		118 (14.5)
	Non ACS, *n* (%)	90 (44.3)	103 (50.7)	87 (42.7)	106 (52.2)		386 (47.5)
Treatment	Primary revascularization within 50 days, *n* (%)	51 (25.1)	46 (22.7)	48 (23.5)	38 (18.8)	0.475*	183 (22.5)
Biomarkers	eGFR, ml/min/1.73 m^2^, median (q1-q3)	63.1 (49.5–72.5)	64.9 (50.5–77.3)	62.3 (48.8–74.4)	64.6 (48.1–80.0)	0.395^†^	63.3 (49.2–75.3)
	hs-CRP, mg/l, median (q1-q3)	3.5 (1.5–13.5)	4.0 (1.7–12.4)	4.0 (1.6–9.9)	4.4 (1.8–16.0)	0.712^†^	4.0 (1.7–13.3)
	BNP, pg/ml, median (q1-q3)	96.0 (40.0–310.0)	106.0 (35.0–334.0)	107.0 (34.0–319.0)	78.5 (29.0–320.0)	0.679^†^	97.0 (34.0–316.0)
	Maximum TnT release >0.01 ng/ml, *n* (%)	113 (55.7)	101 (49.8)	120 (58.8)	96 (47.3)	0.076*	430 (52.9)

MI, myocardial infarction; ACEI, angiotensin converting enzyme inhibitor; ARB, angiotensin receptor blocker; ASA, acetylsalicylic acid; UAP, unstable angina pectoris; NSTEMI, non ST-elevation myocardial infarction; STEMI, ST-elevation myocardial infarction; Non ACS, non acute coronary syndrome; eGFR, estimated glomerular filtration rate; hs-CRP, high sensitivity C-reactive protein; BNP, brain natriuretic peptide; TnT, troponin T.

* Chi–Squared test.

^†^ Kruskal–Wallis test.

**Table 2:** Frequencies of outcome.

Outcome	Year	Total population, *n* (%)	TnT + population, *n* (%)
All-cause death	1	94 (11.6)	80 (18.6)
	2	127 (15.6)	96 (22.3)
	7	314 (38.6)	203 (47.2)
Cardiac death	1	63 (7.7)	57 (13.3)
	2	81 (10.0)	68 (15.8)
MI	1	81 (10.0)	69 (16.0)
	2	140 (17.2)	112 (26.0)
	7	186 (22.9)	136 (31,6)
Stroke	1	15 (1.8)	8 (1.9)
	2	25 (3.1)	13 (3.0)
	7	50 (6.2)	25 (5.8)
All-cause death/MI/Stroke	1	156 (19.2)	126 (29.3)
	2	236 (29.0)	175 (40.7)
	7	392 (48.2)	250 (58.1)
Cardiac death/MI/Stroke	1	133 (16.4)	109 (25.3)
	2	203 (25.0)	155 (36.0)

### 3.1. P1NP and adverse outcomes in the total population

**Figure 1:**
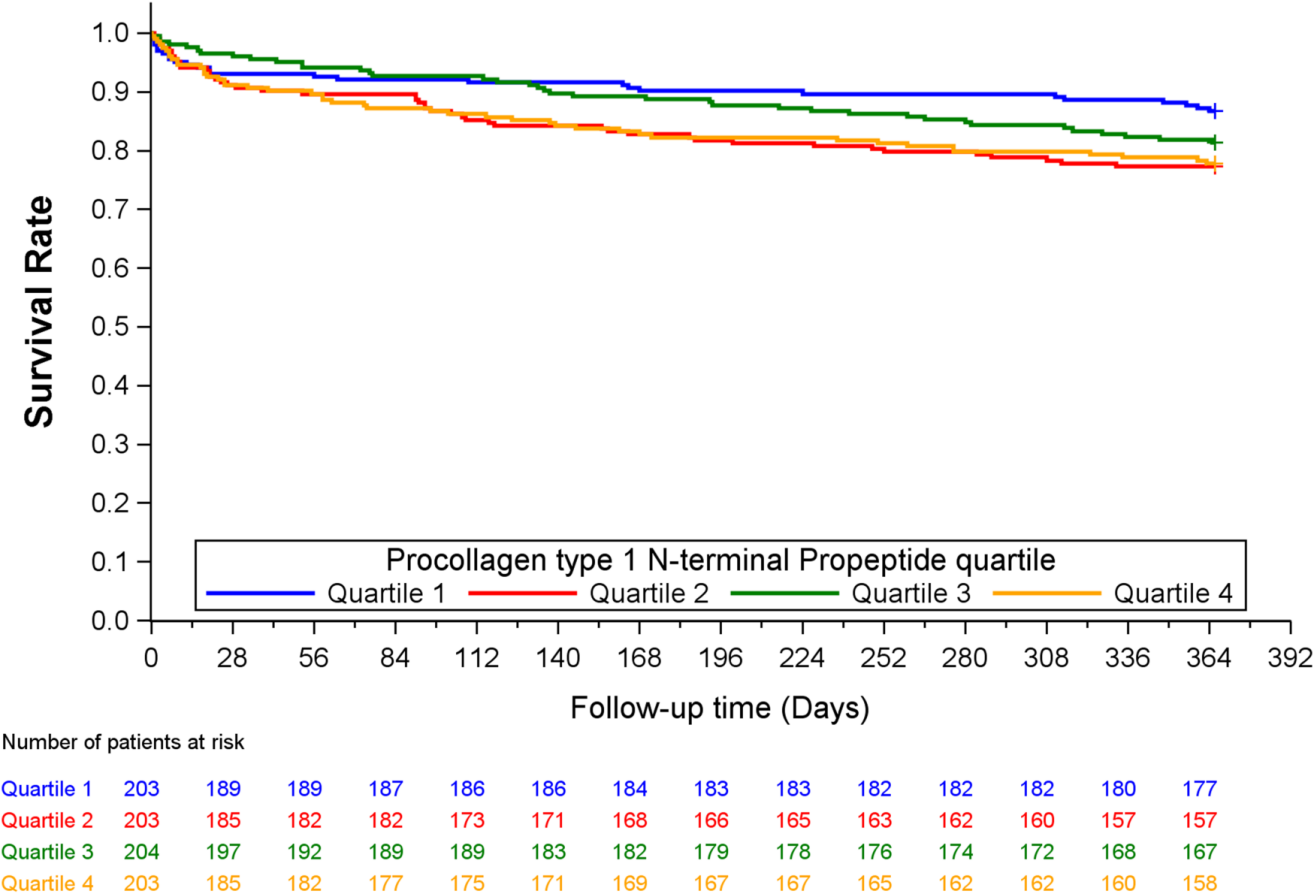
Kaplan–Mier plot of the combined endpoint of all-cause death, new MI or stroke at 1 year, by quartiles of P1NP.

Quartiles of P1NP was significantly associated with the combined endpoint of all-cause death, new MI or stroke at 1-year follow-up in multivariable Cox analysis, HR (Q4 vs. Q1) of 1.82 (95% CI: 1.12–2.98, *p* = 0.017), with consistent significance in testing for both model change and equality of HR. Kaplan–Meier plot for this endpoint is shown in Figure 1. There was also a significant association with the combined endpoint at 1-year in univariable cox analysis (*p* = 0.021), although not in testing for equality of hazards ratios. For the combined outcome of cardiac death, new MI or stroke at 1 year follow-up there was an association found in both univariable and multivariable Cox analysis (*p* = 0.050 and 0.043, respectively), although it did not reach statistical significance for equality of HR in either case. The same was observed for the association with all-cause death at 1-year follow-up in both univariable and multivariable cox analysis (*p* = 0.030 and 0.044, respectively) without significance in the analysis of equality of HR. No association could be found for the individual endpoints of MI, stroke, or cardiac death, nor for the combined endpoints or all-cause death at other time-points. Detailed results of the Cox analyses are given in Table 3. For the endpoint of stroke at 1 year there were too few events for analysis by Cox regression.

**Table 3:** Cox analyses in the total population.

Time	Endpoint	Univariable cox		Multivariable cox*	
		HR (Q4 vs. Q1)	*p*-value^†^	HR (Q4 vs. Q1)	*p*-value^†^
1 year	All-cause death/MI/Stroke	1.75 (1.09–2.82)	0.021^‡^	1.82 (1.12–2.98)	0.017
	All-cause death	1.96 (1.07–3.60)	0.030^‡^	1.92 (1.02–3.63)	0.044^‡^^,^^§^
	MI	1.39 (0.71–2.71)	0.339^‡^	1.13 (0.57–2.23)	0.723^‡^^,^^§^
	Stroke	NA		NA	
	Cardiac death/MI/Stroke	1.69 (1.00–2.84)	0.050^‡^	1.72 (1.02–2.91)	0.043^‡^
	Cardiac death	1.73 (0.85–3.54)	0.133^‡^	1.82 (0.89–3.72)	0.102^‡^^,^^§^
2 year	All-cause death/MI/Stroke	1.27 (0.88–1.84)	0.207^‡^	1.39 (0.94–2.05)	0.098^‡^^,^^§^
	All-cause death	1.50 (0.91–2.48)	0.113^‡^	1.50 (0.89–2.55)	0.130^‡^^,^^§^
	MI	1.05 (0.64–1.72)	0.842^‡^	1.06 (0.64–1.77)	0.822^‡^^,^^§^
	Stroke	2.16 (0.65–7.18)	0.208^‡^	2.50 (0.75–8.30)	0.135^‡^^,^^§^
	Cardiac death/MI/Stroke	1.21 (0.80–1.81)	0.367^‡^	1.33 (0.87–2.02)	0.189^‡^^,^^§^
	Cardiac death	1.27 (0.69–2.33)	0.446^‡^	1.20 (0.64–2.27)	0.573^‡^^,^^§^
7 year	All-cause death/MI/Stroke	0.97 (0.73–1.29)	0.838^‡^	1.05 (0.77–1.41)	0.769^‡^^,^^§^
	All-cause death	0.99 (0.72–1.37)	0.970^‡^	1.08 (0.77–1.51)	0.660^‡^^,^^§^
	MI	0.99 (0.65–1.51)	0.977^‡^	1.03 (0.66–1.59)	0.912^‡^^,^^§^
	Stroke	0.96 (0.43–2.18)	0.928^‡^	1.10 (0.49–2.50)	0.818^‡^^,^^§^

MI, myocardial infarction.

* Model adjusted for significant confounding variables among age, sex, diabetes mellitus, hypertension, current smoking, dyslipidaemia, prior heart disease, heart failure, prior medication, index diagnosis, primary revascularization within 50 days, BNP, eGFR, CRP and peak TnT.

^†^ *p* value for HR Q4 vs. Q1.

^‡^ *p*-value for equality of hazard ratios >0.05.

^§^ *p*-value for model improvement >0.05. NA—Not applicable due to few events. Regression not possible.

### 3.2. P1NP and adverse outcomes in the TnT positive subpopulation

Baseline characteristics of the TnT positive subpopulation are given in Supplementary Table S1. There was a significant difference across quartiles of P1NP with more previous use of ACEI/ARB and diuretics in higher quartiles of P1NP.

P1NP significantly discriminated patients with and without the combined endpoint of all-cause death, new MI or stroke at 1-year in this subpopulation (AUROC = 0.56, *p* = 0.04). An association with the combined endpoint was seen with significance in univariable cox (*p* = 0.028), but without reaching significance for equality of HR. There was no other significant association between P1NP and any endpoint at any time, neither in univariable nor in multivariable testing. Details of the cox analyses are shown in Supplementary Table S2.

## 4. Discussion

We found a significant association between quartiles of P1NP and the combined outcome of all-cause death, new MI or stroke within 1 year. There was also a borderline significant association with the combined outcome of cardiac death, new MI or stroke, as well as with the individual endpoint of all-cause death, at 1-year follow-up. In the TnT-positive subpopulation, there was a borderline significant association with all-cause death at 1-year follow-up in univariable cox analysis. There was no association with other outcomes at any time-points in this material.

As for the predictive role of P1NP in chest pain, no reports are available in emergency patients with acute onset of symptoms suggestive of ACS. Only two small series have so far been reported in stable patients referred for elective diagnostic procedures. One was a series of 46 patients referred for stress thallium-201 single photon emission computed tomography where no association was found between P1NP and the number of diseased vessels, and no change was found in the biomarker level after revascularization (9). In the other study, 43 patients with normal coronary angiography who developed chest pain during an exercise ECG were found to have higher P1NP levels than those without symptoms (10). As both type of patients and outcome measures in these two series differ from our acute chest pain population, no direct comparison of the results is possible.

Studies in patients with MI reveals heterogenous results concerning P1NP and prediction of adverse outcome. An association of increased P1NP levels with reinfarction and CV death was reported in one series of acute MI (14). Other studies also found an association with surrogate endpoints of adverse outcome (LV volume, reduced EF, infarct size and other risk factors including dyslipidemia and higher GRACE score) (15, 16). However, in two other MI series P1NP was neither associated with CV death nor with hospitalization for heart failure or LV remodeling (17, 18). Two series have investigated temporal change in P1NP following MI, both showing elevations in P1NP compared to controls, but no study has investigated change in P1NP as a predictor of adverse events (15, 19). In MI with acute heart failure, P1NP seems not to predict CV mortality or change in EF (20, 21).

There are numerous studies investigating P1NP as a risk marker in chronic HF. Indeed, there is some evidence for P1NP being elevated in HF and decreases with effective treatment (22). P1NP was found to correlate with cardiac function in some series, however others did not find any association with HF development, mortality or hospitalization for HF (23–27). Even though there is some divergence in the literature, several reviews conclude that P1NP is not associated with clinically important endpoints in HF (28, 29).

P1NP has also been investigated in healthy subjects, where several series have shown an association with all-cause mortality and MI, as well as with surrogate endpoints such as CAD risk score and pulse wave velocity (7, 8, 30–32). Two series from an Australian elderly general population showed increased all-cause mortality and increased incidence of MI, but without a similar association to CV-death or to cancer mortality (7, 31). The mechanism of the association of P1NP to mortality and morbidity in that population remains to be elucidated.

The reason for the lack of association with endpoints in our study after two and seven years of follow-up is uncertain, but could potentially reflect that ECM remodeling is of particular pathogenic significance during the first year after ACS. As there may be differences between a MI population and a general population in prior studies (7, 14–18, 31), and we investigated a chest pain population with roughly 50% troponin positive patients, this may also have affected the loss of association. However, this will have to be further evaluated in even larger studies.

The physiology of P1NP in cardiac disease is not fully elucidated. Collagen type 1 is the most abundant ECM protein in the myocardium, conferring tensile strength, while collagen type 3 gives elasticity. The breakdown of collagen by matrix metalloproteinases (MMPs) is regulated by Tissue Inhibitors of MMPs (TIMPs). The ultimate results of either buildup or breakdown of collagens are dependent on the ratio of synthesis to degradation. Degradation is in turn dependent on the ratio of MMPs to TIMPs (3). There is increased production of both type 1 and type 3 collagen in ischemic cardiac disease, and the ratio shifts towards type 1 (33). This would explain some of the changes seen in postinfarction fibrosis with increased stiffness and development of HF. The independent function of P1NP in human physiology is not certain, but experimental research suggests a role in regulation of collagen synthesis and cell adhesion, perhaps especially in embryonic development (34, 35). After reaching circulation, P1NP is rapidly bound by scavenger receptors in liver and metabolized (36, 37). As increased P1NP signifies increased collagen deposition, it could potentially inform decisions about anti-fibrotic treatment in cardiac disease, but the evidence for this is lacking.

A significant difference was seen in the use of ACEI/ARB and diuretics between quartiles of P1NP in our TnT positive subpopulation despite no difference in number of cases with previous history of hypertension or heart failure across the quartiles. This pattern was not seen in the full study population. ACEI is shown to lower levels of P1NP, while different diuretics have variable effects on levels of P1NP (21, 38–42). We assessed both ACEI/ARB and diuretic use in the multivariable analyses, and hence this should not affect the statistical association of P1NP to outcomes in our study. Several other drugs (e.g., anti-osteoporotic drugs, and corticosteroids) may also to some degree influence P1NP levels, as can comorbidities such as fractures and other bone disease (40). We did not further assess the relations of various drugs to P1NP levels and to clinical outcome in our data.

The dynamics of P1NP suggest that blood sampled at different time points may reflect variable phases of disease and healing. Although the sampling time varies in the literature, no sampling time for optimal risk stratification has been defined. In this study, blood samples were taken at hospital admission, as close as possible to symptom onset. Impact of timing on our results remains uncertain. As medication prior to hospital admission could affect levels of P1NP we have included this information in baseline characteristics and in our multivariable analyses. Furthermore, our study patients were enrolled in 2002 to 2003 and were treated according to the guidelines of that time. This raises the question of how more current therapies may influence P1NP levels and its association with clinical outcome. We still believe that from a mechanistic perspective, our study and significant findings are valuable. One potential limitation of our study is the long storage of the blood samples in a biobank before the P1NP analysis. Sample stability in freezer and during multiple freeze-thaw cycles has been demonstrated for P1NP for up to at least 2 years, but no studies have investigated stability for a more extended period of time (40). However, patients were recruited to the study consecutively during a short time period, were stored together under similar conditions (i.e., −8°C) and with similar volumes, so it is assumed that all samples would be equally affected by long-term storage.

Our main finding of a significant association between P1NP and the composite endpoint of all-cause death, MI or stroke within 1 year was backed by consistently significant results in the Cox analyses on all three levels, namely LR test for model change, equality of quartile HR, and HR Q4 compared to Q1. The interpretation was more uncertain for several of the other endpoints as the *p*-value in one or two of the tests were above the significance level. Being an exploratory study not adjusted for multiple comparisons, we chose a conservative approach and interpreted mixed test results as non-significant.

## 5. Conclusions

In our material, P1NP was found to be an independent biomarker significantly associated with adverse clinical outcome in patients admitted to hospital for acute chest pain of suspected coronary origin. This is the first report in the literature on the prognostic value of P1NP in this clinical setting, and the results needs further documentation in independent series. Future studies are warranted in order to clarify the definite role of P1NP as a predictor of adverse outcome in this and other clinical conditions related to cardiovascular disease.

## Data Availability

The raw data supporting the conclusions of this article will be made available by the authors, without undue reservation.
