# Correction: Procollagen type 1 N-terminal propeptide is associated with adverse outcome in acute chest pain of suspected coronary origin

**DOI:** 10.3389/fcvm.2025.1650107

**Published:** 2025-07-07

**Authors:** Thomas Andersen, Thor Ueland, Pål Aukrust, Dennis W. T. Nilsen, Heidi Grundt, Harry Staines, Volker Pönitz, Frederic Kontny

**Affiliations:** ^1^ Department of Anesthesiology, Stavanger University Hospital, Stavanger, Norway; ^2^ Department of Clinical Science (K2), University of Bergen, Bergen, Norway; ^3^ Research Institute of Internal Medicine, Oslo University Hospital Rikshospitalet, Oslo, Norway; ^4^ Thrombosis Research Centre (TREC), Department of Clinical Medicine, UiT—The Arctic University of Norway, Tromsø, Norway; ^5^ Institute of Clinical Medicine, Faculty of Medicine, University of Oslo, Oslo, Norway; ^6^ Section of Clinical Immunology and Infectious Diseases, Oslo University Hospital Rikshospitalet, Oslo, Norway; ^7^ Department of Cardiology, Stavanger University Hospital, Stavanger, Norway; ^8^ Department of Clinical Science, University of Bergen, Bergen, Norway; ^9^ Department of Pulmonology, Stavanger University Hospital, Stavanger, Norway; ^10^ Sigma Statistical Services, Balmullo, United Kingdom; ^11^ Drammen Heart Centre, Drammen, Norway

**Keywords:** biomarkers, acute coronary syndomes, mortality/survival, risk factors, extracellular matrix (ECM), collagen

Correction on Procollagen type 1 N-terminal propeptide is associated with adverse outcome in acute chest pain of suspected coronary origin By Andersen T, Ueland T, Aukrust P, Nilsen DWT, Grundt H, Staines H, Ponitz V, Kontny F (2023). Front Cardiovasc Med. 10:1191055. doi. 10.3389/fcvm.2023.1191055

Affiliation “Department of Clinical Science (K2), University of Bergen, Bergen, Norway” was omitted from the paper. This affiliation has now been added for author Thomas Andersen.

The original version of this article has been updated.

