# Supplementary figures and images for: Procollagen type 1 N-terminal propeptide is associated with adverse outcome in acute chest pain of suspected coronary origin
Source: Front Cardiovasc Med. 2023 Sep 4;10:1191055. doi: 10.3389/fcvm.2023.1191055 (PMC10507464; doi:10.3389/fcvm.2023.1191055)

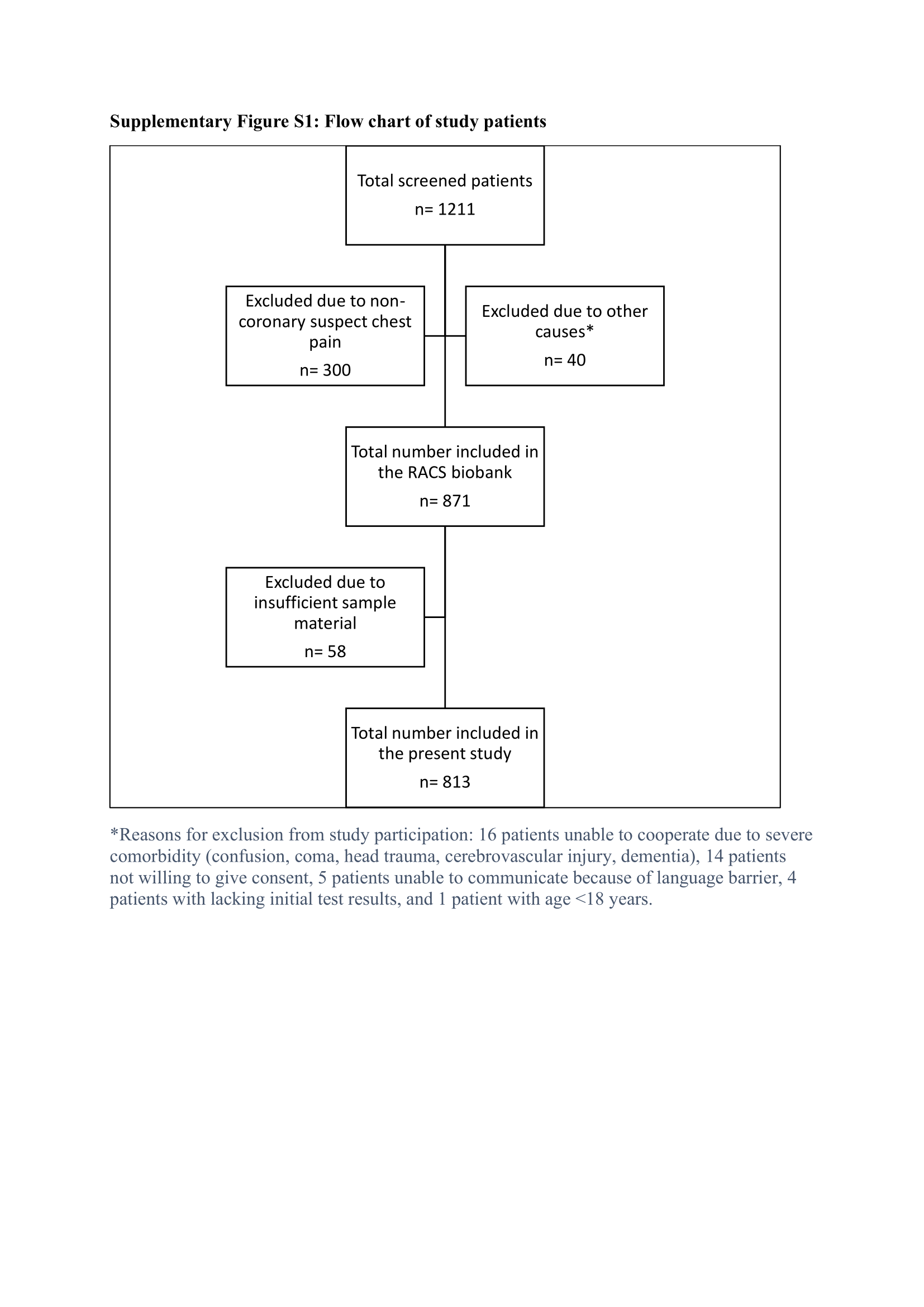

Supplement: Supplementary file 1 [file Image1.tiff]
