# Supplementary material for: Procollagen type 1 N-terminal propeptide is associated with adverse outcome in acute chest pain of suspected coronary origin
Source: Front Cardiovasc Med. 2023 Sep 4;10:1191055. doi: 10.3389/fcvm.2023.1191055 (PMC10507464; doi:10.3389/fcvm.2023.1191055)
Supplement: Supplementary file 2 [file Table1.docx]

Table S1: Baseline characteristics of the TnT positive subpopulation

| **Characteristics** | | **P1NP (ng/mL)** | | | | | |
| --- | --- | --- | --- | --- | --- | --- | --- |
|  |  | **Quartile 1** | **Quartile 2** | **Quartile 3** | **Quartile 4** | **p-value** | **Total** |
|  |  | **n = 107** | **n = 107** | **n = 109** | **n = 107** |  | **n = 430** |
|  |  | **2.50 - 31.60** | **31.70 - 42.30** | **42.40 - 53.70** | **53.80 - 135.5** |  | **2.50 - 135.5** |
| Demographics | Age, years, median (q1-q3) | 74.8 ( 61.7 - 82.4 ) | 74.5 ( 63.6 - 82.9 ) | 73.7 ( 62.6 - 81.5 ) | 74.6 ( 63.4 - 83.2 ) | 0.910† | 74.5 ( 62.9 - 82.6 ) |
|  | Male, n (%) | 68 ( 63.55 ) | 70 ( 65.42 ) | 80 ( 73.39 ) | 66 ( 61.68 ) | 0.278* | 284 ( 66.05 ) |
| Comorbidities | Diabetes mellitus type I or II, n (%) | 19 ( 17.76 ) | 19 ( 17.76 ) | 18 ( 16.51 ) | 17 ( 15.89 ) | 0.978* | 73 ( 16.98 ) |
|  | Hypertension, n (%) | 46 ( 42.99 ) | 43 ( 40.19 ) | 43 ( 39.45 ) | 48 ( 44.86 ) | 0.841* | 180 ( 41.86 ) |
|  | Current smoking, n (%) | 31 ( 28.97 ) | 28 ( 26.17 ) | 36 ( 33.03 ) | 28 ( 26.17 ) | 0.643* | 123 ( 28.60 ) |
|  | Dyslipidaemia, n (%) | 52 ( 48.60 ) | 55 ( 51.40 ) | 41 ( 37.61 ) | 46 ( 42.99 ) | 0.179* | 194 ( 45.12 ) |
|  | Prior MI or angina, n (%) | 58 ( 54.21 ) | 58 ( 54.21 ) | 58 ( 53.21 ) | 68 ( 63.55 ) | 0.378* | 242 ( 56.28 ) |
|  | Prior heart failure, n (%) | 28 ( 26.17 ) | 31 ( 28.97 ) | 33 ( 30.28 ) | 44 ( 41.12 ) | 0.095* | 136 ( 31.63 ) |
| Medication Prior to admission | Statins, n (%) | 28 ( 26.17 ) | 33 ( 30.84 ) | 36 ( 33.03 ) | 35 ( 32.71 ) | 0.679* | 132 ( 30.70 ) |
|  | Betablocker, n (%) | 31 ( 28.97 ) | 36 ( 33.64 ) | 32 ( 29.36 ) | 37 ( 34.58 ) | 0.743* | 136 ( 31.63 ) |
|  | ACEI/ARB, n (%) | 30 ( 28.04 ) | 35 ( 32.71 ) | 36 ( 33.03 ) | 51 ( 47.66 ) | 0.017* | 152 ( 35.35 ) |
|  | Diuretics, n (%) | 25 ( 23.36 ) | 34 ( 31.78 ) | 37 ( 33.94 ) | 51 ( 47.66 ) | 0.002* | 147 ( 34.19 ) |
|  | ASA, n (%) | 32 ( 29.91 ) | 32 ( 29.91 ) | 39 ( 35.78 ) | 44 ( 41.12 ) | 0.245* | 147 ( 34.19 ) |
| Index diagnosis | UAP, n (%) | 16 ( 14.95 ) | 16 ( 14.95 ) | 20 ( 18.35 ) | 22 ( 20.56 ) | 0.528* | 74 ( 17.21 ) |
|  | NSTEMI, n (%) | 56 ( 52.34 ) | 61 ( 57.01 ) | 55 ( 50.46 ) | 62 ( 57.94 ) |  | 234 ( 54.42 ) |
|  | STEMI, n (%) | 34 ( 31.78 ) | 29 ( 27.10 ) | 31 ( 28.44 ) | 23 ( 21.50 ) |  | 117 ( 27.21 ) |
|  | Non ACS, n (%) | 1 ( 0.93 ) | 1 ( 0.93 ) | 3 ( 2.75 ) | 0 ( 0.00 ) |  | 5 ( 1.16 ) |
| Treatment | Primary revascularisation within 50 days, n (%) | 43 ( 40.19 ) | 46 ( 42.99 ) | 41 ( 37.61 ) | 36 ( 33.64 ) | 0.544* | 166 ( 38.60 ) |
| Biomarkers | eGFR, ml/min/1.73m^2^, median (q1-q3) | 62.9 ( 48.3 - 74.7 ) | 64.1 ( 47.3 - 79.8 ) | 61.7 ( 45.0 - 74.5 ) | 58.3 ( 40.5 - 76.2 ) | 0.481† | 61.6 ( 45.0 - 75.4 ) |
|  | hs-CRP, mg/L, median (q1-q3) | 5.3 ( 2.3 - 16.5 ) | 4.3 ( 1.8 - 11.7 ) | 5.9 ( 2.3 - 16.1 ) | 6.7 ( 2.5 - 27.0 ) | 0.193† | 5.7 ( 2.2 - 17.2 ) |
|  | BNP, pg/mL, median (q1-q3) | 163.0 ( 43.0 - 446.0 ) | 147.0 ( 53.0 - 466.0 ) | 194.0 ( 58.0 - 416.0 ) | 206.0 ( 47.0 - 571.0 ) | 0.641† | 176.0 ( 51.0 - 480.0 ) |
| * Chi-squared test. † Kruskal-Wallis test. Significant test in bold. Abbreviations: MI - Myocardial Infarction; ACEI - Angiotensin Converting Enzyme Inhibitor; ARB - Angiotensin Receptor Blocker; ASA - Acetylsalicylic acid; UAP - Unstable Angina Pectoris; NSTEMI - Non ST-elevation Myocardial Infarction; STEMI - ST-elevation Myocardial Infarction; Non ACS - Non Acute Coronary Syndrome; eGFR - estimated Glomerular Filtration Rate; hs-CRP - High sensitivity C-Reactive Protein; BNP - Brain Natriuretic Peptide | | | | | | | |
