# Supplementary material for: Procollagen type 1 N-terminal propeptide is associated with adverse outcome in acute chest pain of suspected coronary origin
Source: Front Cardiovasc Med. 2023 Sep 4;10:1191055. doi: 10.3389/fcvm.2023.1191055 (PMC10507464; doi:10.3389/fcvm.2023.1191055)
Supplement: Supplementary file 3 [file Table2.docx]

Table S2: Cox analyses in the TnT positive subpopulation

| **Time** | **Endpoint** | **Univariable Cox** | | **Multivariable Cox*** | |  |
| --- | --- | --- | --- | --- | --- | --- |
|  |  | **HR (Q4vsQ1)** | **p-value†** | **HR (Q4vsQ1)** | **p-value†** |  |
| 1 year | All-cause death/MI/Stroke | 1.81 (1.07 - 3.06) | 0.028 ‡ | 1.51 (0.87 - 2.62) | 0.139 ‡§ |  |
|  | All-cause death | 1.76 (0.93 - 3.34) | 0.084 ‡ | 1.54 (0.79 - 3.03) | 0.207 ‡§ |  |
|  | MI | 1.84 (0.88 - 3.87) | 0.108 ‡ | 1.22 (0.57 - 2.60) | 0.608 ‡§ |  |
|  | Stroke | NA |  | NA |  |  |
|  | Cardiac death/MI/Stroke | 1.74 (0.98 - 3.08) | 0.057 ‡ | 1.48 (0.83 - 2.64) | 0.179 ‡§ |  |
|  | Cardiac death | 1.48 (0.71 - 3.09) | 0.301 ‡ | 1.32 (0.63 - 2.79) | 0.462 ‡§ |  |
| 2 year | All-cause death/MI/Stroke | 1.34 (0.86 - 2.07) | 0.199 ‡ | 1.25 (0.78 - 1.99) | 0.356 ‡§ |  |
|  | All-cause death | 1.37 (0.77 - 2.42) | 0.283 ‡ | 1.18 (0.64 - 2.16) | 0.594 ‡§ |  |
|  | MI | 1.17 (0.67 - 2.04) | 0.574 ‡ | 1.00 (0.56 - 1.79) | 0.999 ‡§ |  |
|  | Stroke | 2.21 (0.40 - 12.05) | 0.361 ‡ | NA |  |  |
|  | Cardiac death/MI/Stroke | 1.25 (0.79 - 2.00) | 0.342 ‡ | 1.02 (0.62 - 1.66) | 0.943 ‡§ |  |
|  | Cardiac death | 1.11 (0.58 - 2.12) | 0.750 ‡ | 1.03 (0.53 - 2.00) | 0.931 ‡§ |  |
| 7 year | All-cause death/MI/Stroke | 1.09 (0.76 - 1.58) | 0.630 ‡ | 0.92 (0.63 - 1.36) | 0.687 ‡§ |  |
|  | All-cause death | 1.24 (0.83 - 1.85) | 0.296 ‡ | 0.99 (0.65 - 1.50) | 0.944 ‡§ |  |
|  | MI | 1.04 (0.64 - 1.71) | 0.868 ‡ | 0.92 (0.54 - 1.54) | 0.736 ‡§ |  |
|  | Stroke | 0.74 (0.21 - 2.61) | 0.636 ‡ | 0.77 (0.22 - 2.72) | 0.681 ‡§ |  |
| *Model adjusted for significant confounding variables among age, sex, diabetes mellitus, hypertension, current smoking, dyslipidaemia, prior heart disease, heart failure, prior medication, index diagnosis, primary revascularization within 50 days, BNP, eGFR, CRP and peak TnT. † p value for HR Q4vsQ1. ‡ p-value for equality of hazard ratios > 0.05. § p-value for model improvement > 0.05. NA - Not Applicable due to few events. Regression not possible. Abbreviations: MI - Myocardial Infarction | | | | | |  |
|  |  |  |  |  |  |  |
|  |  |  |  |  |  |  |
|  |  |  |  |  |  |  |
|  |  |  |  |  |  |  |
